# A Resource-Efficient, High-Dose, Gamified Neurorehabilitation Program for Chronic Stroke at Home: Retrospective Real-World Analysis

**DOI:** 10.2196/69335

**Published:** 2025-07-10

**Authors:** Spencer A Arbuckle, Anna Sophie Knill, Michelle H Chan-Cortés, Gabriela Rozanski, Anastasia Elena Ford, Louis T Derungs, John W Krakauer, Naveed Ejaz, David Putrino, Jenna Tosto-Mancuso, Meret Branscheidt

**Affiliations:** 1 MindMaze, Lausanne, Switzerland; 2 Rehabilitation Engineering Laboratory, ETH Zurich, Gloriastrasse 39, GLC, Zurich, 8092, Switzerland, +41413996700; 3 Lake Lucerne Institute, Vitznau, Switzerland; 4 Abilities Research Center, Icahn School of Medicine at Mount Sinai, New York, NY, United States; 5 Department of Neurology, Johns Hopkins University, Baltimore, MD, United States; 6 Department of Neuroscience, Johns Hopkins University, Baltimore, MD, United States; 7 Department of Physical Medicine and Rehabilitation, Johns Hopkins University, Baltimore, MD, United States; 8 The Santa Fe Institute, Santa Fe, NM, United States; 9 Center for Neurology and Rehabilitation, Cereneo, Vitznau, Switzerland; 10 Department of Health Sciences and Technology, ETH Zurich, Zurich, Switzerland

**Keywords:** chronic stroke, neurorehabilitation, telerehabilitation, high-dose therapy, real-world evidence, home-based therapy, gamified technologies, asynchronous training

## Abstract

**Background:**

Accumulating evidence and medical guidelines recommend high-dose neurorehabilitation for recovery after stroke. The reality, however, is that most patients receive a fraction of this dose, with therapist availability and costs of delivery being major implementational barriers.

**Objective:**

This study aimed to explore a potential solution by conducting a retrospective analysis of a real-world enhanced clinical service that used gamified self-training technologies at home under remote therapist supervision.

**Methods:**

Data from 17 patients who completed a 12‐18 week full-body, high-dose neurorehabilitation program entirely at home were analyzed. Program delivery relied primarily on patients training independently (asynchronously) with the MindMotion GO (MindMaze) gamified-therapy solution. Accompanying telerehabilitation training sessions with a therapist occurred weekly while therapists used a web application to continuously monitor and manage the program remotely. Effectiveness of the program was assessed through measured active training time, a measure that more closely reflects delivered dosage as opposed to scheduled dose. Patient recovery was evaluated with standardized impairment and functional clinical measures and patient self-reported outcome measures. Finally, a cost model was computed to evaluate the resource efficiency of the program.

**Results:**

Patients maintained high training adherence throughout the program and reached an average total active training time of 39.7 (SD 21.4) hours, with the majority delivered asynchronously (mean 82.2%, SD 10.8%). Patients improved in both upper-limb (Fugl-Meyer Upper Extremity, mean 6.4, SD 5.1; *P*<.001) and gait and balance measures (Functional Gait Assessment, mean 3.1, SD 2.6; *P*<.001; Berg Balance Scale, mean 6.1, SD 4.4; *P*<.001). Overall, the program was viewed very favorably among patients who completed a post-program survey, with 73.7% (14/19) of respondents being satisfied or very satisfied, while 63.2% (12/19) of respondents reported subjective improvements in physical abilities. Per-patient therapist costs approximated US $338, representing a resource-efficient alternative to delivering the same dose via one-on-one in-person training sessions (US $1903).

**Conclusions:**

This work demonstrates effective high-dose neurorehabilitation delivery via gamified therapy technologies at home. The approach shows that training time can be successfully decoupled from therapist-presence without compromising adherence, outcomes, or patient satisfaction over an extended program period. Given growing concerns over therapist availability and increasing health care costs, this resource-efficient approach can help achieve medical guidelines and complement existing clinic-based approaches.

## Introduction

Stroke is a critical global health issue, leaving many individuals with enduring disability [1] and placing an immense economic burden on health care systems. Rehabilitation is pivotal in fostering recovery and restoring functional independence. However, growing evidence suggests that significantly improving clinical outcomes hinges on the provision of high-dose training. Studies indicate that providing at least 20 hours of training over 10 weeks on top of standard therapy is needed to make a clinically meaningful impact [2-6]. Therefore, the importance and clinical benefits of high-dose training are emphasized in existing and emerging medical guidelines for stroke rehabilitation. In the United States, the American Stroke Association guidelines recommend aerobic training of 60‐300 minutes (over multiple sessions and days) per week in addition to strength and stretching exercises [7], while in the United Kingdom, the recent National Institute for Health and Care Excellence guidelines update recommends at least 3 hours of targeted rehabilitation on 5 days per week [8].

However, despite strong evidence supporting the benefits of high-dose rehabilitation, most patients with stroke fail to receive the recommended amount of therapy dose [6,9-12]. During acute inpatient care, patients were found to be active only 13% of their time and only 5.2% in contact with a therapist [13]. A follow-up review nearly a decade later suggested little had changed [14]. Furthermore, following discharge, patients complete less than 20 minutes of upper extremity training during approximately 45-minute sessions [15,16]. This issue becomes particularly pronounced in the chronic stage, where observational studies in outpatient settings have shown that overall training doses remain low, averaging just one purposeful leg or arm movement per minute [17,18].

Thus, there is a stark gap between the evidence supporting high-dose neurorehabilitation versus what is delivered in traditional standard-of-care. Key implementational barriers that contribute toward this gap are human resource constraints (eg, therapist cost and availability) and poor patient adherence and engagement [2,4,19,20]. Therapist availability in particular is only expected to worsen, with a recent national US survey indicating that 66.6% of therapists intend to leave the profession within the coming year [21].

To address these barriers, numerous studies have tried to dissociate therapist time and outpatient infrastructure from therapy itself, either by incorporating technologies, particularly robotics, into in-clinic training programs [22]; using home-based training with or without the aid of additional technologies [23]; incorporating a training model with a single therapist used to deliver care to multiple patients simultaneously [24,25]; or a combination of approaches [26]. However, none of these approaches have fully overcome implementational barriers: they often fail to free up therapist time, are costly, and have low patient adherence and engagement. To further complicate the matter, studies generally fail to demonstrate that scheduled training time equates to actual delivery of high-dose training—the vast majority do not report on actual therapy time delivered [27].

Among newer solutions, gamified rehabilitation technologies have emerged as promising tools for delivering structured, engaging, and measurable training. These systems typically use motion tracking, game-based tasks, and real-time performance feedback to support motor training and encourage adherence. In stroke rehabilitation, gamified technologies have been reported to produce outcomes comparable to those achieved through conventional therapy [11,28]. A promising feature of these systems is that they offer additional flexibility in how and where training is delivered. Their compatibility with remote care models means that patients can engage in therapy at home, either self-directed according to their own schedule (asynchronously) or under remote therapist supervision (synchronous telerehabilitation), offering a potential path to overcoming key implementational barriers to high-dose rehabilitation. Indeed, a recent clinical trial demonstrated that home-based telerehabilitation delivered via gamified technologies was as effective as in-clinic therapy for patients with stroke; however, its remote implementation still relied on therapist supervision for half of the total scheduled training time [29]. Whether patients can deliver the majority of training independently, using gamified technologies in a minimally supervised asynchronous format, remains relatively unexplored. Such an approach could provide a more scalable pathway to deliver high-dose rehabilitation that aligns with the growing constraints on therapist time and system resources. Demonstrating the feasibility of patients achieving the bulk of their training dose independently is a critical next step.

Thus, in this real-world enhanced clinical service and retrospective analysis, we investigated the extent to which implementational barriers related to delivering high-dose neurorehabilitation can be addressed with a technological approach. Our primary objective was to determine whether gamified training and monitoring technologies can be used to successfully deliver high-dose neurorehabilitation for chronic stroke survivors at home. As part of this aim, we report on therapy adherence and clinical changes experienced by patients. By setting a soft target that 75% of the training dose was to be delivered through patient self-training (asynchronous) with the remaining 25% relying on synchronous telerehabilitation with the therapist, we attempted to dissociate therapeutic dose delivery from therapist availability.

Training was delivered with the aid of a gamified training technology (MindMotion GO) that enabled full-body training of the upper limbs, hand, trunk, and lower limbs. Monitoring components of the gamified technology system were used to track actual training times and to ensure that therapists were able to consistently oversee patient progress and uphold the quality and success of the training. We performed separate evaluations of patient adherence, clinical effectiveness of training, patient satisfaction, and overall resource and cost efficiency of the program.

## Methods

### Patients

Patients with stroke were identified from the Mount Sinai Abilities Research Center Clinical Program registry. Patients were informed of the enhanced clinical service and offered the option to participate in high-dose neurorehabilitation, which was offered from October 2022 to August 2023. The program enrolled patients who were ≥22 years of age; had a first stroke episode ≥3 months prior; presented with either arm weakness and/or difficulty with balance; were able to follow multistep commands; and showed willingness and ability to commit to the program length and the weekly training dose accompanied with telerehabilitation sessions. Patients inappropriate for the program included those with plegia of the affected upper limb, severe pain, or severe cognitive or physical challenges that made it difficult for them to participate in neurorehabilitation.

A total of 26 patients with chronic stroke (defined as having experienced their first stroke ≥6 mo prior) were enrolled in the program. Of these, 23 began training (Figure S1 in Multimedia Appendix 1), but 6 either dropped out or were lost to follow-up. Specifically, 1 patient completed the training but declined to attend the second set of clinical evaluations; 3 experienced unrelated medical changes that prevented further participation; and 2 voluntarily withdrew from the program. Data from the 17 patients (8 women; mean age of 54.8 y, SD 14.1 y; time from first stroke episode: mean 5.4 y, SD 4.7 y; Table 1) who completed the training program and underwent both sets of clinical assessments were analyzed retrospectively. As this was a real-world clinical implementation, the sample size for analysis was limited by patient availability, clinical capacity to provide care, and the availability of clinical service data. There were no significant differences regarding chronicity, age, or impairment (tested with the baseline Fugl-Meyer Upper Extremity and Berg Balance Scale measures) between patients who completed the training program and those who did not, and no adverse events were reported during the program.

**Table 1.** Baseline characteristics for analyzed patients.

Characteristics	Values (N=17)
Age (years), mean (SD)	54.8 (14.1)
Years post stroke, mean (SD)	5.4 (4.7)
Sex, n (%)	
Male	9 (52.9%)
Female	8 (47.1%)
Stroke type, n (%)	
Ischemic	9 (52.9%)
Hemorrhagic	8 (47.1%)
Affected hemisphere, n (%)	
Left	6 (35.3%)
Right	11 (64.7%)

### High-Dose Telerehabilitation Training Program

**Figure 1.**
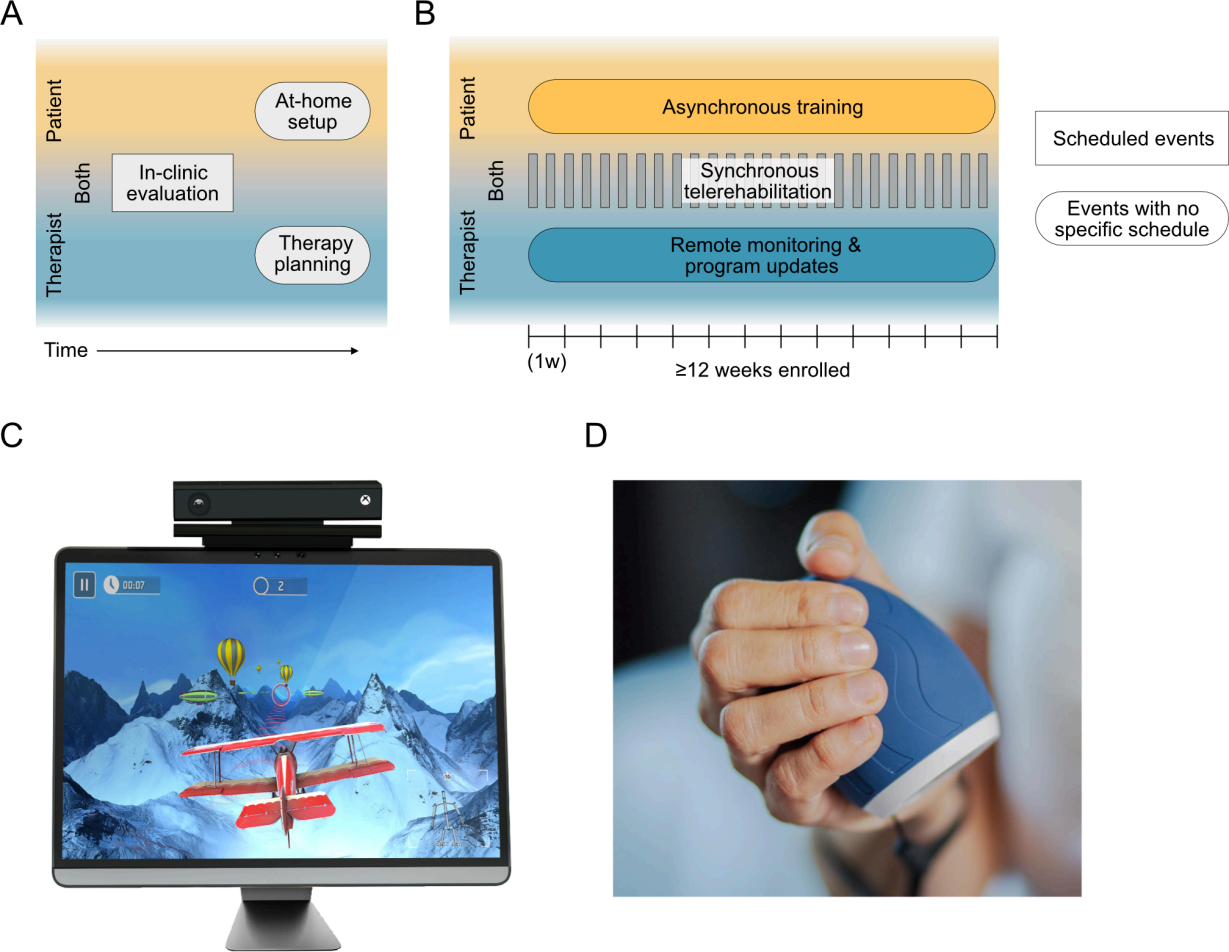
Design and technologies of the home-based training program. Patients were enrolled into a home-based training program that consisted of self-directed (asynchronous) training sessions performed by the patient each week, complemented by multiple weekly sessions directly supervised by a physical therapist (synchronous telerehabilitation). (**A**) Prior to the program start, patients were evaluated in-clinic by a physical therapist. The therapist would then create a training program for the evaluated patient. Patients set up the training system (MindMotion GO and Izar) at home. (**B**) During the training program, synchronous telerehabilitation sessions were scheduled as per therapist and patient availability, whereas asynchronous training (performed by the patient) or remote monitoring (conducted by the therapist) could be completed at any time. Patients additionally underwent an in-clinic assessment battery prior to the program starting and upon program completion to evaluate the clinical efficacy of the training program (not shown). (**C**) The MindMotion GO telerehabilitation device uses camera-based motion tracking to control gamified training activities displayed on a television screen. (**D**) The Izar, a therapeutic device to train hand function, being grasped in a hand.

All patients were assigned to a single-arm treatment group involving a high-dose, home-based training program (Figure 1A–B). For the purposes of this work, we consider a high-dose program to be one that achieves a total dosage above 20 hours scheduled time, as these doses are generally considered to deliver outcomes above the minimum clinically important difference [2,5,29]. Overall, the program goal was to deliver 36 hours of training during the program. The target dose was consistent with a previous large telerehabilitation study that showed significant improvements in upper-limb impairment (42 h [29]). In contrast, training in this program was not limited to one body area [30-32] and could be achieved with multiple effectors (ie, upper-limb, hand, trunk, and lower-limb).

Training dose was delivered via synchronous telerehabilitation and asynchronous training (Table 2). Patients were given the freedom to achieve the 36 hours of training dose over a period of 12-18 weeks and were given a high-level goal to train for at least 120 minutes per week. Ultimately, the exact program duration for each patient was dependent on their availability and desire to continue. Consistent with in-person rehabilitation practice, patients could take short breaks away from the program (these breaks were incorporated in the analysis; see section “Binning Training Data into Weeks” in the Methods). Patients were also free to continue additional regular rehabilitation services.

**Table 2.** Suggested weekly schedule.

Training type	Planned session duration (min)	Planned sessions per week (#)	Expected training per session (min)	Expected training per week (min)
Asynchronous	at patient discretion	at patient discretion	at patient discretion	≥90
Synchronous	30	1‐3	15	30‐45
Total				≥120

#### Synchronous Sessions

These telerehabilitation sessions were guided directly by a physical therapist who was present via video conferencing. The therapist was free to schedule 30-minute synchronous sessions with a patient at a frequency determined by their clinical judgment. The content of the sessions included gamified training using the MindMotion GO system (see section “Full-Body Gamified System for Asynchronous Training” in the Methods) under visual observation of the therapist and check-in conversations between the patient and the therapist to discuss training goals, training adjustments, feedback, etc.

#### Asynchronous Sessions

These were self-directed training sessions where patients performed gamified training in a remote environment (ie, at home). In contrast to synchronous training, asynchronous sessions did not include a therapist present. Patients had the ability to independently choose when and for how long to train asynchronously, and the ability to skip or repeat prescribed activities. At least 75% of a patient’s total dose was expected to be delivered asynchronously.

Asynchronous training content for each patient was determined by their supervising therapist, who tailored their training program by selecting gamified activities on the MindMotion GO based on the patient’s disease severity, therapeutic goals, preferences, and their physical capacity, with the range of motion calibrations defining the playable range of each activity. This calibration ensured that the gameplay was within the patient’s safety envelope (to minimize pain and adverse events such as risk of falls), and was updated as needed. The gamified activities targeted one or more areas of the following: hand, upper limbs, trunk, and/or lower limbs.

### Full-Body Gamified System for Asynchronous Training

A commercially available technology (MindMotion GO; Food and Drug Administration Class II) was used to deliver full-body gamified training. The MindMotion GO is a real-time, motion capture–based neurorehabilitation therapy technology (Figure 1C) that (1) enabled therapists to set up and track training, (2) enabled patients to perform asynchronous training sessions, (3) provided teleconsultation capabilities for synchronous training and check-ins, and (4) captured performance measures during gameplay that allowed for the quantification of the dose and nature of training delivered to each patient. At the time of this enhanced clinical service, the MindMotion GO catalog included 36 gamified activities designed to train specific movements relevant to neurorehabilitative therapy (see Table S1 in Multimedia Appendix 1).

Patients’ body and limb kinematics were captured by accompanying optical hardware sensors (Kinect and LeapMotion), which were used to drive the mechanics of the gamified activities. An additional peripheral device (Izar, MindMaze; Figure 1D) was used to capture fine-grained forces generated during hand and wrist control and use them for gameplay.

A range of metrics related to a patient’s training performance was captured in real-time and uploaded to a web platform (Companion, MindMaze). Therapists used this web platform to remotely set up or adjust therapy plans and to track training progress.

Equipment was shipped to a patient’s home, where it was connected to a television. All but one patient successfully installed the system without requiring in-person assistance from the therapy team. Customer and technical support (via telephone or video-call) was provided for the duration of the program.

### Quantification of Dose and Nature of Training

We analyzed training data from the MindMotion GO device to assess program feasibility and patient behavior, including training time, duration, and movements trained.

#### Quantifying Dose Delivered

Active training time (ATT), defined as the time spent engaging in gamified activities (excluding setup, calibration, or pauses), was used as a measure of training dose delivered.

#### Binning Training Data Into Weeks

Our primary focus was on training program dosage (ie, hours of ATT) rather than program duration (ie, weeks in the program). However, to investigate training behavior over time, training data were organized into “training weeks”, defined as consecutive 7-day periods starting on the patient’s program start date and ending at their exit assessment. Training weeks could vary across patients and if a patient missed training in a week (ie, zero ATT), it was excluded (5.8% of all total 346 enrolled weeks). For clarity, we use “trained weeks” in figures in the Results section, acknowledging that these may not reflect consecutive weeks.

#### Assessing Training Consistency

We examined whether patients’ asynchronous training behavior changed over time using ordinary least squares regression. A positive or negative beta coefficient indicated whether the proportion of ATT trained asynchronously tended to increase or decrease across weeks, respectively. The magnitude of a coefficient represents the weekly rate of change (eg, a coefficient of 0.001 is +1% increase per week). The beta coefficient for each patient is presented in the Results section.

#### Analyzing Training Schedules

We tested if patients had idiosyncratic but nevertheless reliable training schedules. To assess patients’ training schedules, we calculated the distribution of their total asynchronous ATT across each hour of each day of the calendar week. For each patient, data from even- and odd-numbered training weeks were binned separately. We then computed Pearson correlation between training schedules for even- and odd-numbered weeks within patients to measure schedule reliability. To quantify the uniqueness of each patient’s schedule across the cohort, we computed the average Pearson correlations between their even- and odd-week schedules and those of all other patients’ schedules. The within-patient and across-patient correlations are presented in the “Results” section.

### Capturing Clinical Outcomes and Patient Subjective Experiences

We quantified the effect of training on patients’ impairment and function. As training was delivered full-body, we captured a wide range of functional outcome measures and patient reported outcome measures (PROMs) to quantify training-related changes across multiple effectors. A comprehensive list of all outcome measures is available in Multimedia Appendix 1 (Table S2 and Table S5).

Clinical outcome measures and PROMs were captured at the beginning and the end of the program. The clinical outcome measures were assessed in person at the Abilities Research Center by a physical therapist who was not their supervising therapist to retain blindness. PROMs were completed by patients through web surveys captured via Research Electronic Data Capture (REDCap) tool [33,34]. A custom survey was administered to all enrolled patients at the end of the program, regardless of training completion, to gather information on their experiences, satisfaction, motivation, and perceived improvements.

### Cost Analysis

We conducted a simplified cost analysis, comparing physical therapist resourcing costs for two scenarios: (1) this program, with training delivered via a combination of asynchronous and synchronous sessions and (2) a scenario where the entire dose is delivered through direct supervision. The median hourly salary for a physical therapist was sourced from the US Bureau of Labor Statistics 2023 [35], which excludes fringe benefits (eg, medical insurance). Therapist costs for both scenarios were calculated by multiplying the total training hours by the median hourly salary (in USD), adjusted for the percentage of training delivered under supervision.

### Statistical Analysis

All analyses and statistical tests were performed using R (R Core Team, version 4.3.1 [36-39]). Values presented in the results are the average across patients (plus or minus the SD). For Pearson correlation values, we first Fisher z-transformed the correlations and calculated the mean and SD. The mean and SD were then transformed back to correlations and reported according to convention. To compare if the proportion of asynchronous ATT systematically changed for all patients across the program duration, we used a 2-sided *t* test to compare the beta coefficients to 0 (see section “Assessing Training Consistency” in the Methods). To compare if patients had idiosyncratic asynchronous training schedules, we used a 2-sided paired *t* test to compare the Fisher z-transformed correlations from within and across-patient comparisons of their training schedules (see section “Analyzing Training Schedules” in the Methods). We set the significance level to *P*=.05 for these statistical tests.

For the analysis of clinical improvements, changes from baseline to program end were tested using paired 2-sided *t* tests. As these tests involved multiple comparisons without any specified a priori hypotheses, we adjusted the significance level by dividing by the number of clinical outcomes examined, which was 12 (ie, Bonferroni correction). For clarity, we report uncorrected *P* values in the text. Note that not all 17 patients were evaluated on all assessments, and therefore, the degrees of freedom varied across the clinical outcomes.

Results of the exit survey are reported as a proportion of total survey respondents, which differs from patients who completed the program. We chose to present the exit survey results for all respondents to ensure all views were included, irrespective of whether a patient officially completed the program. A total of 19 patients completed the exit survey, of whom 16 completed the program and 3 did not (2 dropped out and 1 was lost to follow-up).

### Ethical Considerations

All data were collected as a part of routine clinical care at the Abilities Research Center at the Icahn School of Medicine at Mount Sinai. Institutional review board approval for retrospective analysis of clinical data was obtained from the Mount Sinai Program for Protection of Human Subjects (Icahn School of Medicine at Mount Sinai, New York, United States; STUDY-21‐00345). This approval includes a consent waiver, allowing for the retrospective analysis of the data for purposes beyond routine clinical care without requiring additional patient consent. Patients received no monetary compensation. Prior to analysis, all data were deidentified by the clinical staff at the Abilities Research Center.

## Results

### Long-Term, High-Dose Training Can Be Achieved for Patients With Chronic Stroke in the Home

A retrospective analysis was carried out on 17 patients with chronic stroke who completed high-dose neurorehabilitation. Overall, the goal was for patients to receive a minimum of 36 hours of ATT (see section “High-Dose Telerehabilitation Training Program” in the Methods), delivered entirely at home and spread across all major motor functions (ie, upper limbs, hand, trunk, and lower limbs). Training was delivered using a gamified therapy solution (MindMotion GO with Izar), through a combination of the patients following prescribed training plans on their own (self-directed asynchronous training) supplemented by weekly training sessions with a therapist being present via video conferencing (therapist-directed synchronous telerehabilitation). Therapists were able to remotely define and alter training plans and maintain oversight of the program using a web-based application for patient monitoring (Companion).

**Figure 2.**
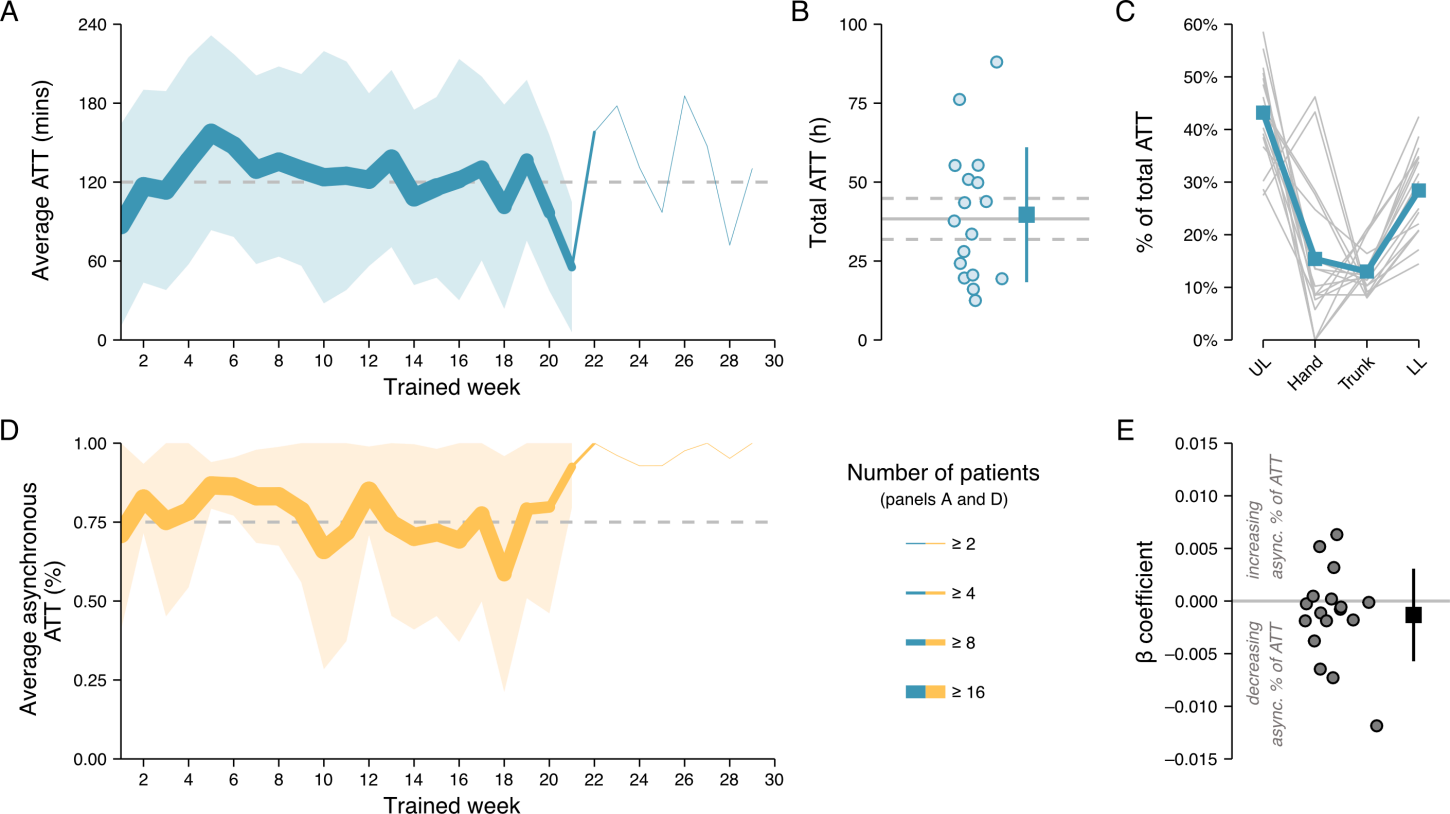
Training dose. (A) Active training time (ATT) per week averaged across patients. The thickness of the line represents the number of patients who remained in the program by that training week. The shaded area indicates the SD above and below the average (for weeks with n>3), and the dashed line indicates the target weekly dosage of 120 minutes. (B) The total ATT dosage achieved at program end. Each dot corresponds to one patient. The box indicates the average and the vertical line indicates the SD above and below the average. The solid horizontal line indicates the total expected dosage if each patient achieved 120 minutes ATT per week they were training in the program, with the dashed horizontal lines reflecting the SD around the average expected dose (patients completed a varying number of training weeks in the program). (C) Proportion of total ATT that patients spent training their upper limbs (UL), hands, trunk, or lower limbs (LL). Individual patient data are depicted as thin lines, with the average across patients shown as a thicker line. (D) The proportion of weekly ATT that was achieved asynchronously, averaged across patients. Similar formatting as in (A). (E) Quantifying patients’ tendency to increase, decrease, or maintain their ratio of asynchronous-to-synchronous ATT across training weeks (see Methods). On average, patients maintained a steady proportion of asynchronously achieved ATT across weeks.

Training duration and consistency were high. Patients trained an average of 120.51 (SD 52.47) minutes per week for 19.18 (SD 3.24) weeks (Figure 2A), with all patients exceeding the intended minimum duration of 12 weeks. Overall, patients achieved an average cumulative training dose of 39.67 (SD 21.37) hours, slightly surpassing the program goal of 2 hours of ATT per week (Figure 2B). This cumulative training dose was full body, with a slight tendency toward upper limb training (mean 43.20%, SD 9.06% of total ATT) compared to the lower limb (mean 28.40%, SD 8.27%), hand (mean 15.40%, SD 14.09%) and trunk (mean 13.01% SD 4.25%; see Figure 2C). For a breakdown of dosage per each of the 27 possible movements that could be trained, see Figure S2 in Multimedia Appendix 1. Altogether, these results demonstrate the feasibility of hybrid asynchronous and synchronous programs to deliver actual high-dose training distributed across the full body.

It is important to note that the ATT dose measure used in this analysis is a closer representation of the dose actually delivered in an intervention, as compared to the scheduled dose that is almost exclusively reported in other high-dose neurorehabilitation studies [5,10,29,40,41]. Furthermore, this dose was delivered in addition to any other rehabilitation received by patients. Indeed, 12 out of the 17 patients also reported receiving care outside the program (eg, physiotherapy, occupational therapy, or general exercise), highlighting the program’s complementarity to standard-of-care neurorehabilitation. We did not observe any significant differences in program adherence measures between patients who did versus did not report participating in additional therapies (Table S3 in Multimedia Appendix 1).

Despite the extended program duration of approximately 19 weeks, patients reported high levels of satisfaction. In an exit survey, 73.7% of respondents (14/19) indicated they were either very satisfied or satisfied with the program (Figure S3A in Multimedia Appendix 1). In addition, patients expressed strong motivation to continue training beyond the program length, with 57.9% (11/19) reporting they were very motivated or motivated to continue (Figure S3B in Multimedia Appendix 1). This was particularly apparent in one patient who trained for 29 weeks, far surpassing the official program length.

### Majority of Program Dose Was Delivered Without the Presence of a Therapist

Therapists scheduled synchronous telerehabilitation sessions with patients as per their clinical discretion to ensure compliance with the overall program. However, the goal was for patients to achieve much of their training dose asynchronously. On average, each patient achieved 34.18 (SD 21.72) hours of training asynchronously, representing 82.19% (SD 10.81%) of their total ATT (Figure 2D). This constituted the majority of their overall dose and exceeded the 75% target.

Patients continued to complete most of their training asynchronously as the program progressed (Figure 2D), with no systematic change in the ratio between synchronous and asynchronous training dose from week to week (Figure 2E; mean $\hat{\beta }$=−1.3e−3, SD 4.4e-3, 2-sided *t* test versus 0: *t*_16_=−1.24, *P*=.23).

**Figure 3.**
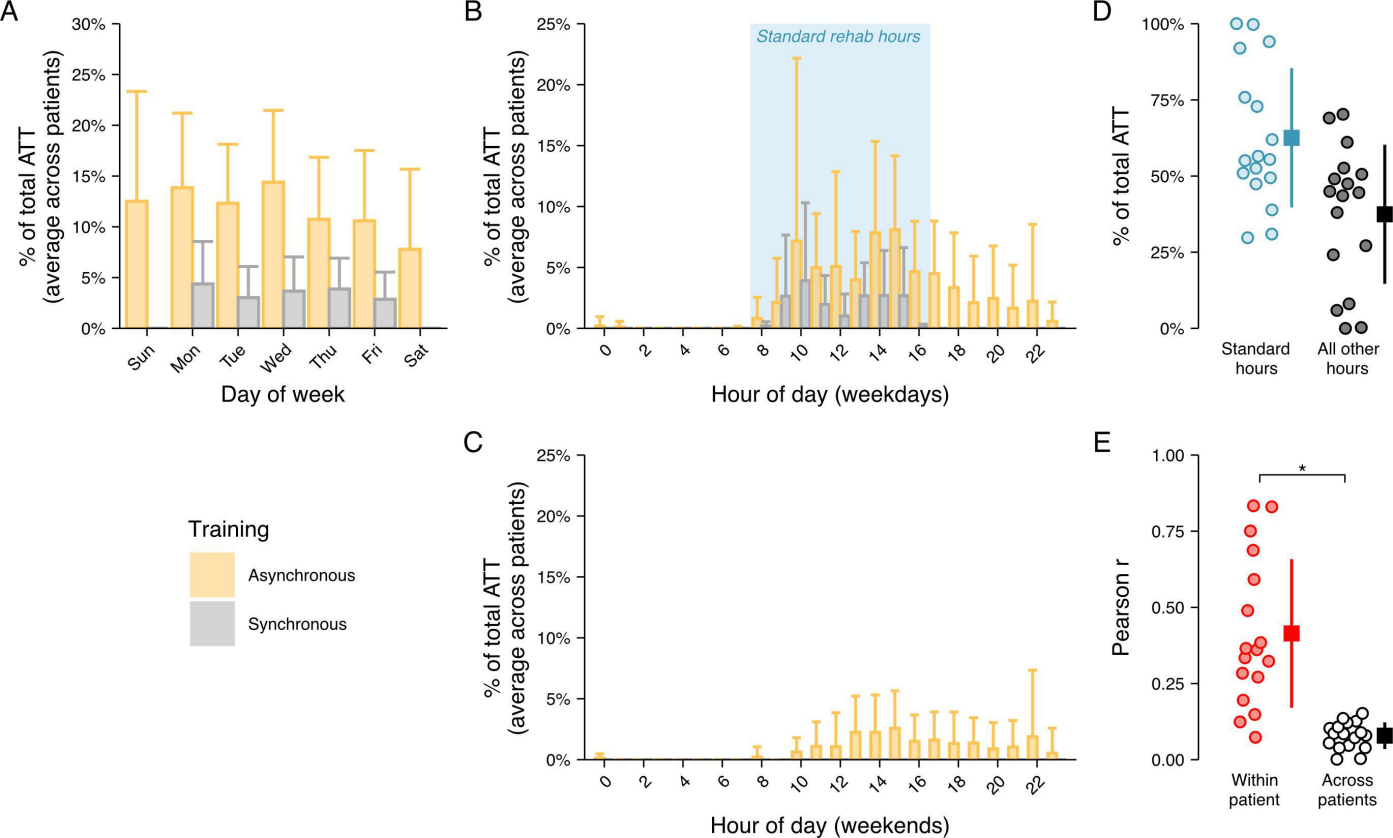
Training distribution across days of the week and times of the day. (A) Bar chart depicting the proportion of total ATT (averaged across patients) binned according to the day of the week. Synchronous training times were only logged during the weekdays. Error bars reflect the SD across patients. (B-C) Bar charts depicting the proportion of total ATT (averaged across patients) binned according to the starting hour of the training session for training done on weekdays (B) and weekends (C). Similar formatting as in A. (D) Percent of total ATT that occurred during standard working hours (ie, when the therapist was available for synchronous telerehabilitation sessions; box in B) versus that which occurred during all other hours. Each dot corresponds to one patient. The boxes indicate the average across patients and the vertical lines indicate the SDs above and below the averages. (E) Quantifying the uniqueness of patients’ average weekly asynchronous training profiles (see “Methods” section). Each dot in the within-patient grouping reflects a patient’s consistency of their weekly training profile, and each dot in the across patients grouping reflects the average correlation between that patient’s training profile and the training profiles of all other patients. The boxes and vertical lines are formatted the same as in D. *denotes significant difference between within-patient and across patient-correlations (2-sided paired t test, *P*<.01).

All synchronous sessions were delivered during regular 8 AM-5 PM (Monday-Friday) working hours Figure 3A-C In contrast, asynchronous training offered patients the flexibility of training at any time, decoupled from restrictions of therapist availability. Indeed, a substantial proportion of each patient’s total ATT (mean 37.45%, SD 22.84%; mean 15.70, SD 12.29 h) was logged outside of working hours and on weekends (Figure 3D).

Patients’ own schedules imposed restrictions on when they were able to train. Interestingly, while the majority of patients remained relatively consistent in their overall training schedules from week to week (mean within-patient Pearson *r*=0.46, SD 0.34, see Figure 3E), the training schedules were highly individual (mean across-patients Pearson *r*=0.08, SD 0.04, see Figure 3E; 2-sided paired *t* test versus within-patient schedule reliability: *t*_16_=−4.74, *P*<.001).

Taken together, these results demonstrate that patients remained highly adherent to the home-based training program even if training was largely delivered asynchronously.

### Patients Showed Significant Improvements in Gait, Balance, and Upper-Limb Function

Next, we investigated the effect of the high-dose training on patients’ recovery and self-reported well-being. As the delivered training was full-body, we captured a wide range of standardized upper limb, gait and balance, and physiological measures to quantify training-related changes in impairment and function across multiple effectors (see section “Capturing Clinical Outcomes and Patient Subjective Experiences” in the Methods). All measures were obtained at the beginning and end of the training program.

Patients demonstrated positive improvements in three core impairment and functional measures for multiple effectors (see Table 3), namely the Fugl-Meyer Upper Extremity assessment for upper limb impairment, (FM-UE; mean +6.41, SD 5.09, n=17, *P*<.001), the Berg Balance Scale for generalized balance and transfer stability (BBS; mean +6.07, SD 4.43, n=15, *P*<.001), and the Functional Gait Assessment (FGA; mean +3.07, SD 2.55, n=15, *P*<.001) for postural stability and walking. Among these, both the FM-UE and BBS were above the Minimal Detectable Change (MDC) for chronic stroke (MDC=3.2 and 4.66 points, respectively [42,43]), whereas the FGA was not (MDC=4.2 points [44]). These clinical improvements occurred irrespective of whether patients participated in additional therapies (see Table S4 in Multimedia Appendix 1). Although there were positive improvements across most other captured measures, they did not cross thresholds for either statistical significance or minimum detectable change (see Table S2 in Multimedia Appendix 1). These positive improvements in standard clinical measures were complemented by 63.2% of respondents (12/19) self-reporting subjective improvements in their physical abilities and well-being (Figure S3C in Multimedia Appendix 1). Additional patient-reported outcome measures are reported in Table S5 in Multimedia Appendix 1.

Overall, these results demonstrate that patients receiving a high-dose training program at home showed significant improvements in outcome measures related to full-body impairment and function, with these improvements reinforced by patients’ self-reported measures.

**Table 3.** Core clinical outcomes.

Clinical measure^a^	Number of patients evaluated	Potential range	Evaluation before program start		Evaluation at program end		∆ score (end-baseline scores)		
			mean (SD)	Range	mean (SD)	Range	mean (SD)	*t* (df)*^b^*	*P* value^c^
FM-UE^d^	17	0‐66	31.71 (21.22)	8‐65	38.12 (20.03)	14‐66	6.41 (5.09)	5.20 (16)	8.8×10^−5^
ARAT^e^	17	0‐57	19.00 (21.45)	0‐57	21.53 (23.29)	1‐57	2.53 (4.20)	2.48 (16)	2.5×10^–2^
BBS^f^	15	0‐56	38.67 (11.18)	14‐55	44.73 (9.15)	20‐56	6.07 (4.43)	5.30 (14)	1.1×10^–4^
FGA^g^	15	0‐33	9.87 (6.03)	1‐23	12.93 (6.20)	1‐28	3.07 (2.55)	4.66 (14)	3.7×10^–4^
5xStS^h^	15	>0 s	22.00 (10.89) s	8‐45	17.17 (7.21) s	8‐34	−4.83 (6.25) s	−3.00 (14)	9.6×10^–3^
6minWT^i^	14	≥0 ft	579.64 (324.83) ft	120‐1175	635.00 (371.46) ft	130‐1420	55.36 (86.77) ft	2.39 (13)	3.3×10^–2^

a Scores of the complete set of 12 clinical outcomes are presented in Table S2 in Multimedia Appendix 1.

b The *t* values and associated significance for two-sided paired *t* tests comparing scores at program end to baseline.

c Statistical significance is reported as uncorrected values. A Bonferroni correction for 12 comparisons was applied when evaluating significance.

d FM-UE: Fugl-Meyer Upper Extremities

e ARAT: Action Research Arm Test.

f BBS: Berg Balance Scale.

g FGA: Functional Gait Assessment.

h 5xStS: 5 times Stand to Sit.

i 6minWT: 6-minute Walk Test.

### Asynchronous Training Significantly Reduces Costs Associated With Delivery of High-Dose Training

Finally, we computed the personnel costs associated with the delivery of this high-dose training. Based on the current analysis, 39.7 (SD 21.4) hours of cumulative training dose was delivered to each patient on average, with the majority being delivered asynchronously (82.2%, SD 10.8%). Therefore, direct therapist presence was required to deliver only 17.8% of the training dose, and thus the total therapist costs per patient would amount to US $338 (given the median hourly salary for a physical therapist as US $47.94 [35]).

In contrast, the delivery of regular therapy hours relies on direct therapist presence either through in-clinic visits or using teleconferencing tools [45]. Therefore, under a scenario where all training is delivered directly, the costs of delivering 39.7 hours would amount to US $1903—a substantial increase of US $1565 when compared to the current hybrid asynchronous and synchronous program.

## Discussion

### Principal Findings

While the evidence for high-dose training in stroke rehabilitation is accumulating, the real challenge is in its implementation: how to deliver high-dose training effectively and in a resource-efficient way? Here we demonstrate that a gamified neurorehabilitation program can be used to successfully deliver high-dose full-body training to patients with chronic stroke at home. Using a combination of therapist-directed telerehabilitation sessions (synchronous) and patients’ training on their own (asynchronous training), we were able to achieve resource-efficient dose delivery—the program required only a fraction of the therapist’s time compared to traditional one-to-one in-person neurorehabilitation sessions. To our knowledge, this work is the first to demonstrate a significant decoupling of active training dose delivery from therapist presence.

Despite this decoupling, patients in the program showed clinical improvements. Unlike most previous approaches, patients showed positive improvements in all measures related to gait, balance, and upper-limb function, thus addressing that stroke typically results in multilimb deficits. These improvements were noticeable by patients, with 63.2% (12/19) reporting higher physical abilities and overall well-being.

### The Role of Gamified Technology to Deliver High-Dose Training at Home

A critical issue in most neurorehabilitation studies is the lack of transparent reporting between scheduled therapy and what is truly delivered. A recent review identified the scale of the problem, with approximately only 13% of studies reporting on the actual training dose received [27]. When regular therapy is quantified, the actual time on task is a fraction of that scheduled, and is significantly lower than training doses required for recovery [46].

Using technologies has two advantages here: (1) namely as a means of sensitively and accurately quantifying the amount of training dose delivered, while (2) also providing a set of training activities that homogenizes the nature of training received by the patient. Such technologies therefore respond to a growing demand for better documentation and transparency of dose delivery in neurorehabilitation [47].

A key element of this program was the significant use of asynchronous training delivery to achieve a higher dose. Our core mitigation strategy against reductions in patient engagement and adherence [48] was to ensure that the therapist was always in the loop, with the synchronous telerehabilitation sessions serving as a means to review and update the plan of care, but also to ensure patient engagement. In addition, gamified activities, a wide variety of exercises, and user-friendly interfaces were identified as important components [49] and were implemented in the technology used. Our results demonstrate the success of these mitigation strategies, with patients able to self-administer over 80% of the total training dose and maintain consistent engagement.

That patients remained engaged in the program suggests that they could realize a principal advantage of training asynchronously: convenience. Patients were able to train anytime without being restricted by therapist availability. Indeed, here we found that when patients were given a high-level training goal, supplied with tailored training programs delivered through gamified technologies, and provided with therapist check-ins, they used both weekdays and weekends to train which is not possible with conventional in-person neurorehabilitation. Furthermore, patients tended to train with highly idiosyncratic schedules that were most likely driven by unique work, life, and social commitments.

### Comparing Approaches to Deliver High-Dose Training

In previous studies, a variety of different approaches for delivering high-dose training have been investigated, but few are truly scalable in a resource-constrained health care landscape. High-dose approaches that require one-on-one in-clinic therapy sessions are prohibitively expensive, are often limited to urban facilities, and struggle to scale (ie, treat more patients) without significant staffing [5]. Offering one-to-many training can mitigate the staffing and cost concerns but often imposes restrictions on patient scheduling. Supplementing regular therapy with robotic systems imposes high equipment and maintenance costs [50], and the complexity of these devices constrains them to supervised use in-clinic [51].

In comparison, while home-based programs are an attractive alternative, they are not all equal. Programs that rely solely on passive exercise videos may be inexpensive, but they often struggle with poor therapy adherence rates and a limited ability to customize care plans. And while traditional telerehabilitation benefits from direct therapist and patient contact with respect to adherence, it is again dependent on therapist availability [48].

What remains attractive and potentially scalable, however, are home-based programs that blend primarily asynchronous training with synchronous sessions. Such hybrid programs can be delivered through various commercially available gamified training systems, which integrate motion tracking, performance feedback, and game-based physical exercises to enhance motivation and adherence. These systems have the potential to address geographic, economic, and engagement-related implementational barriers to delivering high-dose training. The MindMotion GO, used in this study, exemplifies this class of technologies. It has previously been implemented in supervised, in-clinic settings for individuals with subacute stroke and multiple sclerosis [52], as well as in outpatient telerehabilitation contexts [53], though detailed clinical outcomes in these cases have not been reported. While these earlier implementations demonstrate usability, they do not clarify whether such systems can enable effective, asynchronous high-dose training in real-world, at-home settings–particularly for individuals with chronic stroke. This study addresses this gap by retrospectively evaluating a hybrid telerehabilitation program in which patients completed the majority of their own training asynchronously using a gamified system. Hybrid programs offer a promising solution to many of the known challenges associated with traditional high-dose rehabilitation delivery, including therapist availability and patient scheduling flexibility.

While recent studies have demonstrated the efficacy of providing high-dose training at home [29,30], the full value of these programs was not realized; there were significant requirements on therapist availability (50:50 synchronous vs asynchronous split) and no accompanying cost analysis. The work here extends these results and demonstrates that technology-enabled asynchronous training allows for high training doses to be delivered with limited demands on therapist availability (82% was delivered asynchronously). Despite reduced therapist presence in the program, patient engagement and satisfaction remained high. We also report costs associated with program delivery, which represent a fraction of the resource costs associated with delivering equivalent high-dose training through scaling up one-to-one neurorehabilitation.

### Integrating Asynchronous Training Into Standard-of-Care

For home-based neurorehabilitation programs to gain widespread adoption, it is critical that they integrate into and complement current neurorehabilitation workflows. Here we offer two pragmatic suggestions. For patients that routinely receive in-person outpatient rehabilitation, part of the in-person sessions can be used to review, discuss, and update the plan-of-care for remote asynchronous training. In contrast, patients in more rural settings, who may have limited access to in-person care, could reduce in-person visits with synchronous telerehabilitation sessions, again complemented by training asynchronously. Indeed, following the COVID-19 pandemic, synchronous telerehabilitation in the United States remains a billable service due to public health emergency extensions [54], while new current procedural billing codes have been established by the Center of Medicare & Medicaid Services to provide reimbursement pathways for technologies that enable asynchronous neurorehabilitation services [55,56].

### Challenges of Clinical Implementation

Despite the attractiveness of a high-dose neurorehabilitation at home, a few caveats should be considered. In such programs, there is a tangible concern that the importance of therapists is reduced. We unequivocally disagree with this notion. Therapists are, and will remain, essential for building collaborative relationships with patients that are critical for engagement and recovery [57]. To this end, it is essential that the technologies are designed with therapists’ needs in mind. Care should also be taken to mitigate the risk of therapist fatigue and burnout associated with the specific inherent demands of teleconferencing [58].

Home-based asynchronous training offers significant benefits, but it may not be suitable for all patients. Barriers such as limited physical space, privacy concerns, and the need for technical proficiency can hinder widespread adoption of training technology [59]. With respect to this last item, the relatively young average age of our cohort might suggest higher technical literacy and fewer cognitive impairments, which influences generalizability. In addition, deficits in vision, communication, cognition, or a heightened fall risk may also significantly impact the safety and effectiveness of predominantly asynchronous therapy programs [60,61]. Moreover, some patients have historically preferred in-person therapy [28,59]. Taken together, these points only serve to highlight the critical role of therapists in properly selecting and recommending home-based neurorehabilitation only to appropriate patients.

### Limitations

Despite promising results, this study has several limitations. First, the absence of a control group prevents us from drawing any definitive causal conclusions about the impact of remote high-dose training on clinical improvements. However, establishing causality was not the primary aim. The goal of this work was to assess if high levels of training dose could be delivered via a real-world clinical service, as emerging consensus from animal models [62] and human trials [2] suggests that it results in improved upper-limb and gait and balance outcomes. Indeed, medical guidelines for stroke advocate high-dose training [7,8], but patients often receive much less in the chronic setting, posing an implementation challenge. For example, in the United States, patients with chronic stroke typically receive very little or no rehabilitation [63-66], leading to a continual decline in functioning across 5 years after ischemic stroke, irrespective of age and stroke severity [67]. Thus, while we cannot make causal claims, improvements observed here contrast with the typical experience of patients with chronic stroke.

Second, the small sample size, constrained by patient availability and clinical capacity, may limit the generalizability of the findings. Future studies should address factors such as patient motivation, comorbidities, socioeconomic status, and caregiver support. Furthermore, of the 23 patients that began training in this clinical service, 6 dropped out (see section “Patients” in the Methods). Although only 2 of the 6 dropouts voluntarily withdrew from care, suggesting a low dissatisfaction rate, we cannot rule out the possibility that patient attrition may have influenced the results.

Third, the cost analysis focused exclusively on therapist time, excluding the cost of equipment. This approach was intentional to ensure applicability across different commercially available gamified telerehabilitation systems, which vary in price. Furthermore, a true comparison of technology costs would require documenting the different technologies routinely used as part of standard-of-care (eg, robotics, virtual reality therapies, antigravity support systems, etc.), which was not captured as part of this study.

### Conclusion

This work introduces an asynchronous and gamified technology-driven approach for delivering high-dose training to patients with stroke. We believe this approach has the potential to scale to address therapist shortages economically and effectively. This work, to our knowledge, is the first demonstration of the full potential value of hybrid asynchronous and synchronous high-dose neurorehabilitation programs at home. The program described here is potentially an attractive option to bridge the gap between accumulating scientific evidence and medical guidelines around the need for high-dose training, and the reality of traditional neurorehabilitation today.

## Supplementary material

10.2196/69335 Multimedia Appendix 1 Supporting figures and tables.

## Abbreviations

5xStS: 5 times Sit to Stand Test
6minWT: 6 Minute Walk Test
ARAT: Action Research Arm Test
ATT: active training time
BBS: Berg Balance Scale
FGA: Functional Gait Assessment
FM-UE: Fugl-Meyer Upper Extremity
MDC: Minimal Detectable Change
PROM: patient reported outcome measure
