# Supplementary material for: A Resource-Efficient, High-Dose, Gamified Neurorehabilitation Program for Chronic Stroke at Home: Retrospective Real-World Analysis
Source: JMIR Serious Games. 2025 Jul 10;13:e69335. doi: 10.2196/69335 (PMC12270188; doi:10.2196/69335)
Supplement: Multimedia Appendix 1 [file games-v13-e69335-s001.pdf]

Appendix

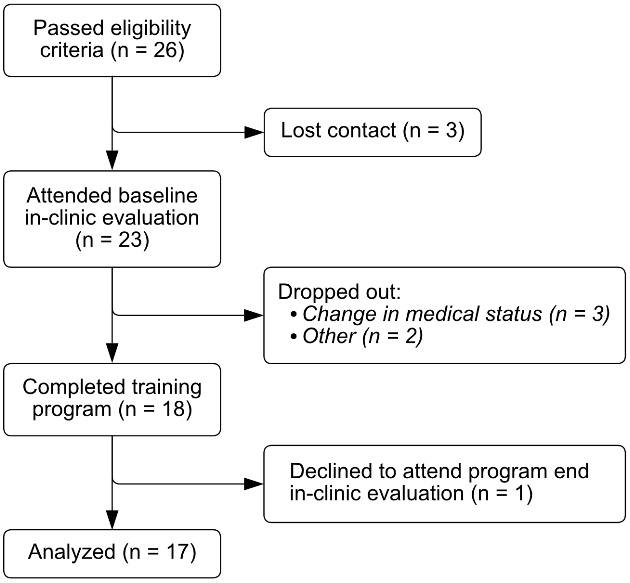

Figure S1. Patient enrollment. Flow of enrolled patients through the program.

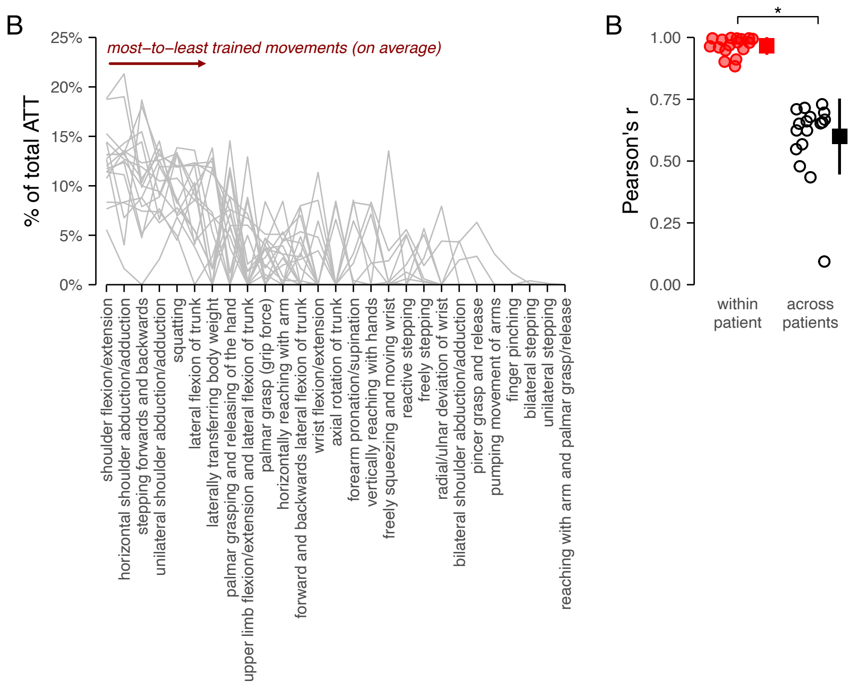

Figure S2. Uniqueness of movements trained across patients. (**A**) Patients' movement training profiles: Proportion of total ATT that patients spent training each of the 27 possible movements covered by games on the MindMotion GO (see Table A1). Each patient's data is plotted as a gray line. (**B**) Quantifying uniqueness of patients' movement training profiles. We used an approach similar to the one in the analysis for Fig. 3E of the manuscript, which demonstrated the uniqueness of patients' training schedules. In this case, we calculated the distribution of patients' total ATT across each of the 27 trained movements, with data from even- and odd-numbered training weeks being binned separately within each patient. We then computed a patient's consistency of their own movement training profile (Pearson's correlation between even and odd week movement profiles; red dots) and compared it to the movement training profiles of all other patients (average correlation between that patient's even and odd week movement training profiles and those of all other patients; white dots). Comparison of the two sets of correlations demonstrated that patients' movement profiles were significantly distinct from one-another (two-sided paired t test comparing the Fisher z-transformed correlations from within and across-patients: $t_{16}=11.94$, $p<0.001$).

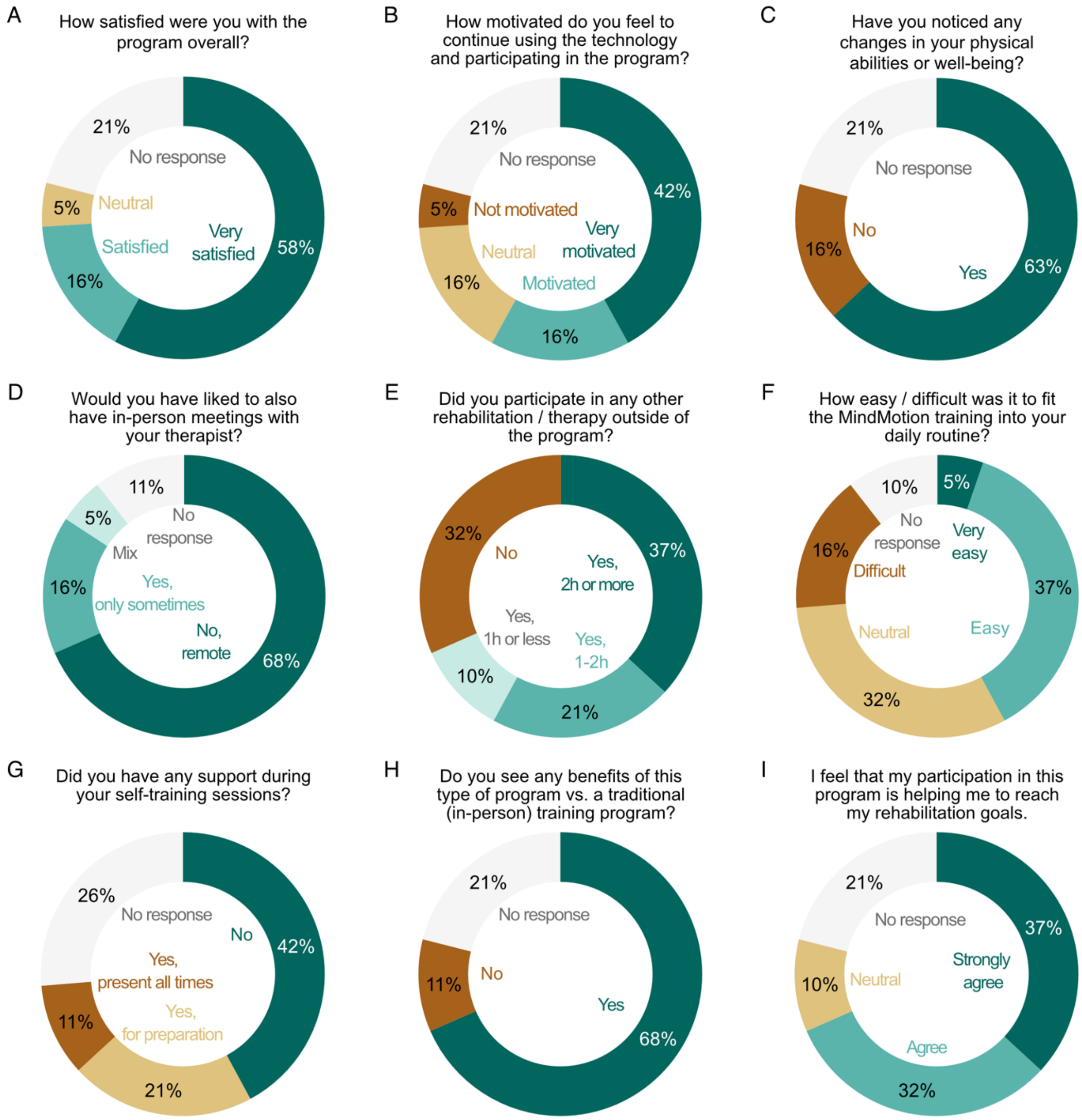

Figure S3. Qualitative exit survey results. Results from the exit survey (n = 19 respondents) about (**A**) satisfaction; (**B**) motivation; (**C**) changes in physical well-being; (**D**) preference of synchronous telerehabilitation vs. in-person sessions; (**E**) participation in additional training; (**F**) ease of fitting the training program into a daily routine; (**G**) support needed during asynchronous training; (**H**) perceived advantage of the home-based training program; and (**I**) patients' perceived ability to reach their rehabilitation goals by participating in this program.

Table S1. Training movements. Movements that patients could train with the MindMotion GO and Izar.

Body region targeted	Movement
Hand / Wrist	Palmar grasp (grip force)
	Palmar grasping and releasing of the hand (3 activities)
	Finger pinching
	Pincer grasp and release
	Freely squeezing and moving wrist
	Wrist flexion/extension (2 activities)
	Radial/ulnar deviation of wrist
	Forearm pronation/supination (3 activities)
Upper Limb	Horizontally reaching with arm
	Vertically reaching with hands
	Reaching with arm and palmar grasp/release
	Unilateral shoulder abduction/adduction
	Bilateral shoulder abduction/adduction
	Horizontal shoulder abduction/adduction
	Pumping movement of arms (bilateral shoulder, elbow, and forearm)
	Shoulder flexion/extension
	Upper limb flexion/extension and lateral flexion of trunk
Trunk	Lateral flexion of trunk
	Axial rotation of trunk
	Forward and backwards lateral flexion of trunk
Lower Limb	Laterally transferring body weight
	Unilateral stepping
	Bilateral stepping
	Stepping forwards and backwards
	Freely stepping
	Reactive stepping
	Squatting

Table S2. Clinical outcomes. Statistical significance is presented as uncorrected values. Asterisks indicate significance after Bonferroni correction (for 12 comparisons). Abbreviations: FM-UE = Fugl-Meyer Upper Extremities assessment; BBS = Berg Balance Scale; FGA = Functional Gait Assessment; ARAT = Action Research Arm Test; 5xStS = 5 times Stand to Sit; 6minWT = 6-minute Walk Test; TUG = Timed Up and Go Test; HR = Heart Rate; BP = Blood Pressure; NIHSS = National Institutes of Health Stroke Scale; BI = Barthel Index.

			**Potential range**	**Eval. at program start**		**Eval. at program end**		**Δ score**		
**Assessment**		***n***	**min - max**	**mean (sd)**	**range**	**mean (sd)**	**range**	**mean (sd)**	***t***	***p***
Functional	FM-UE	17	0 - 66	31.71 (21.22)	8 - 65	38.12 (20.03)	14 - 66	+6.41 (5.09)	5.20	8.8e-5*
	ARAT	17	0 - 57	19.00 (21.45)	0 - 57	21.53 (23.29)	1 - 57	+2.53 (4.20)	2.48	2.5e-2
	BBS	15	0 - 56	38.67 (11.18)	14 - 55	44.73 (9.15)	20 - 56	+6.07 (4.43)	5.30	1.1e-4*
	FGA	15	0 - 30	9.87 (6.03)	1 - 23	12.93 (6.20)	1 - 28	+3.07 (2.55)	4.66	3.7e-4*
	5xStS	15	0 -	22.00 (10.89) sec	8 - 45	17.17 (7.21) sec	8 - 34	-4.83 (6.25) sec	-3.00	9.6e-3
	6minWT	14	0 -	579.64 (324.83) ft	120 - 1175	635.00 (371.46) ft	130 - 1420	+55.36 (86.77) ft	2.39	3.3e-2
	TUG	15	0 -	31.47 (24.62) sec	6 - 80	27.53 (21.33) sec	6 - 88	-3.93 (10.60) sec	-1.44	1.7e-1
Physiological	HR	16	-	78.38 (11.22) bpm	67 - 107	72.81 (10.87) bpm	58 - 96	-5.56 (10.3) bpm	-2.17	4.7e-2
	BP (systolic)	16	-	114.25 (13.24) mmHg	88 - 144	113.31 (13.88) mmHg	95 - 148	-0.94 (13.26) mmHg	-0.28	7.8e-1
	BP (diastolic)	16	-	79.13 (9.70) mmHg	64 - 92	74.44 (7.36) mmHg	62 - 90	-4.69 (8.27) mmHg	-2.27	3.8e-2
Neuro-logical	NIHSS	17	0 - 42	5.41 (3.45)	1 - 11	4.29 (2.91)	1 - 11	-1.12 (1.76)	-2.61	1.9e-2
	BI	17	0 - 100	85.59 (22.70)	15 - 100	87.94 (21.44)	15 - 100	+2.35 (5.04)	1.93	7.2e-2

Table S3. No impact of additional therapy on program adherence. Patients were divided into two groups: those who reported participating in additional forms of physical exercise/therapy (e.g., stretching, exercising, PT, and/or OT) during the program and those who did not. This categorization was based on exit survey responses, detailing the type, frequency, and duration of any additional therapy. Abbreviations: ATT = active training time; sd = standard deviation.

Adherence metric	# patients reported doing additional therapy	# patients with no known additional therapy	Mean YES (sd)	Mean NO (sd)	Welch's 2-sided t-test yes vs. no (p-value)
# weeks trained	12	5	19.583 (3.502)	18.200 (2.588)	0.900 (0.389)
avg. weekly ATT (mins)	12	5	123.267 (51.825)	113.879 (59.578)	0.307 (0.768)
total ATT (hours)	12	5	42.133 (22.944)	33.753 (17.799)	0.809 (0.438)
avg. async. ATT (%)	12	5	82.713% (9.544%)	80.927 (14.623%)	0.252 (0.810)

Table S4. No impact of additional therapy on clinical outcomes. Abbreviations: FM-UE = Fugl-Meyer Upper Extremities assessment; BBS = Berg Balance Scale; FGA = Functional Gait Assessment; sd = standard deviation; Δ = change from program start to program end.

Assessment	# patients reported doing additional therapy	# patients with no known additional therapy	Mean YES Δ (sd)	Mean NO Δ (sd)	Welch's 2-sided t-test yes vs. no Δ (p-value)
FM-UE	12	5	+5.333 (4.418)	+9.000 (6.164)	-1.207 (0.274)
BBS	11	4	+5.455 (4.009)	+7.750 (5.737)	-0.737 (0.501)
FGA	11	4	+2.909 (2.809)	+3.500 (1.912)	-0.462 (0.484)

Table S5. Patient reported outcomes. Abbreviations: GAD-7 = General Anxiety Disorder-7; ISI = Insomnia Severity Index; BRS = Brief Resilience Scale; PROMIS = Patient-reported outcomes measurement information system; SIS = Stroke Impact Scale; EQ-5D-5L = European Quality of Life 5 Dimensions 5 Level Version; PHQ-9 = Patient Health Questionnaire.

		**Potential range**	**Eval. at start**	***Eval. at end***	**Δ score**
**Assessment**	***n***	**min - max**	**mean (sd)**	**mean (sd)**	**mean (sd)**
GAD-7	13	0 / 21	3.8 (4.2)	2.0 (2.3)	-1.8* (2.6)
ISI	13	0 / 28	3.9 (3.5)	3.3 (3.4)	-0.5 (2.9)
BRS	15	1 / 6	3.6 (0.9)	3.8 (0.8)	+0.2 (0.7)
PROMIS (social)	13	8 / 40	14.4 (6.4)	14.3 (6.8)	-0.1 (4.1)
PROMIS (pain)	13	8 / 40	13.3 (5.5)	13.2 (6.5)	-0.1 (7.6)
SIS (strength)	12	0 / 100	38.0 (12.1)	38.0 (17.8)	0.0 (16.2)
SIS (hand)	12	0 / 100	17.9 (33.3)	20.4 (36.3)	2.5 (10.1)
SIS (ADL)	10	0 / 100	51.3 (16.4)	51.8 (19.2)	0.5 (9.1)
SIS (mobility)	11	0 / 100	60.4 (28.2)	67.7 (25.5)	7.3 (14.0)
SIS (communication)	11	0 / 100	80.5 (23.1)	84.1 (20.2)	3.6 (13.0)
SIS (emotion)	10	0 / 100	81.9 (17.6)	83.8 (10.6)	1.9 (8.9)
SIS (memory)	10	0 / 100	81.1 (21.5)	81.8 (20.6)	0.7 (11.5)
SIS (participation)	9	0 / 100	42.7 (26.8)	55.2 (26.5)	12.5 (18.5)
EQ-5D-5L (mobility)	13	1 / 5	3.0 (0.9)	2.8 (0.9)	-0.2 (0.6)
EQ-5D-5L (activities)	13	1 / 5	3.2 (1.1)	3.2 (1.2)	-0.1 (0.5)
EQ-5D-5L (anxiety)	13	1 / 5	1.8 (0.9)	1.6 (0.7)	-0.2 (1.1)
EQ-5D-5L (selfcare)	13	1 / 5	2.4 (1.3)	2.5 (1.2)	0.1 (0.8)
EQ-5D-5L (pain)	13	1 / 5	2.1 (0.9)	1.9 (0.6)	-0.2 (0.7)
EQ-5D-5L (vas)	13	1 / 100	61.9 (25.8)	58.8 (20.0)	-3.2 (24.9)
SIS (recovery)	12	1 / 100	50.8 (20.7)	49.2 (13.8)	-1.7 (19.5)
PHQ-9	13	0 / 27	3.8 (4.2)	3.9 (3.5)	+0.2 (2.8)
